# Comparison of Maternal Histories and Exposures in Children With Isolated Anorectal Malformation Versus Anorectal Malformation With Genitourinary Anomalies

**DOI:** 10.7759/cureus.8762

**Published:** 2020-06-22

**Authors:** Mark A Taylor, Brian T Bucher, Ron W Reeder, Marc Levitt, Jeffrey Avansino, Megan M Durham, Casey M Calkins, Richard Wood, Kaylea Drake, Michael Rollins

**Affiliations:** 1 Department of Surgery, University of Utah, Salt Lake City, USA; 2 Department of Pediatrics, University of Utah, Salt Lake City, USA; 3 Division of Colorectal and Pelvic Reconstructive Surgery, Children's National Hospital, District of Columbia, USA; 4 Division of Pediatric General and Thoracic Surgery, Seattle Children's Hospital, Seattle, USA; 5 Division of Pediatric Surgery, Emory University School of Medicine, Atlanta, USA; 6 Department of Pediatric Surgery, Children's Wisconsin, Milwaukee, USA; 7 Pediatric Colorectal and Pelvic Reconstructive Surgery, Nationwide Children's Hospital, Columbus, USA; 8 Department of Surgery, Primary Children's Hospital, Salt Lake City, USA

**Keywords:** imperforate anus, cloaca, vacterl, consortium, prenatal, anal atresia, counseling, premature, congenital

## Abstract

Introduction

To our knowledge, there are no studies to date that have compared patients with isolated anorectal malformation (ARM) to patients with ARM and an associated genitourinary (GU) malformation despite a possible etiological difference between these two entities. We examined the differences in maternal and prenatal exposures and comorbidities between patients with isolated ARM and patients with ARM and associated GU malformations.

Materials and methods

A retrospective cohort study of children with ARM, enrolled in the Pediatric Colorectal and Pelvic Learning Consortium (PCPLC) between February 2017 and October 2019, was performed comparing those with isolated ARM to those with ARM and associated GU anomalies (GU +/- additional anomalies) as well as to those with ARM and a GU anomaly with no anomaly of any other system (GU-only). We compared the prevalence of prematurity, family history of colorectal disorders, as well as maternal and prenatal comorbidities and exposures between these two cohorts and the isolated ARM cohort.

Results

A total of 505 patients (117 with isolated ARM and 388 with ARM and associated GU anomalies) were enrolled. Of the 388 patients with ARM and associated GU anomalies, 48 had an ARM with a GU anomaly without an anomaly in any other system. There was an increased prevalence of premature births in the GU +/- additional anomalies cohort compared to the isolated ARM cohort (27 vs 14%, p=0.003). This difference was not seen in the GU-only cohort. There was no difference between the cohorts regarding prevalence of family history of ARM or maternal and prenatal comorbidities or exposures.

Conclusions

Patients with an ARM and an associated GU anomaly with or without other congenital anomalies are more likely to be born prematurely compared to patients with an isolated ARM. Parents of these children should be counseled on this increased risk.

## Introduction

Anorectal malformations (ARMs) consist of a spectrum of congenital defects in the lower gastrointestinal (GI) tract ranging from anal stenosis to persistence of a cloaca. While the etiology of ARM likely has a genetic component, factors such as environmental and parental also play a role [1-4]. The non-genetic associations that have been reported include maternal overweight/obesity, prematurity, small for gestational age, parental smoking, family history of ARM, fever during pregnancy, maternal occupational chemical exposures, use of assisted reproductive technology, multiple pregnancies, maternal diabetes, previous miscarriages, and maternal medication exposure during pregnancy including anti-asthma medications, hypnotics, and benzodiazepines [3,5-11].

However, isolated ARM compared to ARM with other associated anomalies, such as genitourinary (GU) malformations, may result from different etiological processes [12,13]. For example, van den Hondel et al. demonstrated that genetic disorders occur twice as frequently in patients with ARM and an upper limb anomaly than in patients with ARM without an upper limb anomaly [12]. Furthermore, Khanna et al. described multiple differences in the possible genetic etiological factors between isolated ARM and ARM with congenital anomalies of other systems [13]. Few studies investigating the risk factors of ARM compared the cohort of patients with isolated ARM to those with ARM and anomalies of other systems. Analyzing these two cohorts as a single population rather than distinct entities may disguise the differences between the two groups of patients and obscure factors that may pose risks to one of the cohorts.

In addition, studies on ARM to date are largely limited by a small sample size due to the rarity of the disease [8]. To overcome the small number of patients that present to any single institution, the Pediatric Colorectal and Pelvic Learning Consortium (PCPLC) was established in 2016. The mission of the consortium is to facilitate multicenter research collaboration to increase the number of patients available for prospective and retrospective studies so that questions related to ARM and other rare pediatric colorectal and pelvic diseases might be definitively answered [14].

We hypothesized that there are significant differences between the prevalence of prematurity, family history of colorectal disorders, and maternal/prenatal comorbidities, as well as exposures between patients born with an isolated ARM and those born with an ARM and associated GU anomaly.

## Materials and methods

A multicenter, retrospective cohort study of children with ARM was conducted, which was evaluated at the enrolling sites of the PCPLC. The University of Utah Institutional Review Board reviewed and approved this research. The PCPLC consists of eight tertiary children’s hospitals enrolling pediatric colorectal patients’ clinical information into a central database [14]. All patients were prospectively entered into the PCPLC centralized patient registry from February 2017 to October 2019. Patients were included in this study if they had a diagnosis of ARM and completed an intake questionnaire. There were no exclusion criteria. Prenatal maternal history was obtained from parent history and available medical records. The abstracted data included patient demographics, patient comorbidities, maternal medical history, and family history. Preterm birth was defined as less than 37 weeks gestational age.

The cohort of patients with isolated ARM was compared to the cohort of patients with ARM and an associated GU anomaly. The patients in the latter cohort may or may not have another anomaly in a separate system in addition to the GU anomaly (GU +/- additional anomalies cohort). The subset of patients from the GU +/- additional anomalies cohort with ARM, an associated GU anomaly, and no anomalies in other systems (GU-only) was also compared to the isolated ARM cohort. GU anomalies were investigated due to their close embryological association with the GI tract, thus decreasing any confounding factors that might result in other congenital anomalies. GU anomalies included any congenital anomalies in the kidney, bladder, ureters, genitalia, or urethra. Other systems with anomalies are cardiovascular, chromosomal anomalies, endocrine, upper GI system, craniofacial, hematologic, musculoskeletal, neurologic, psychiatric, and respiratory. Patient demographics and comorbidities, maternal medical history, and family history were summarized using counts and percentages for nominal variables and using the median and interquartile range for interval variables. Missing data for gestational age at birth were excluded from the analysis and reported as unknown. Missing data for race and ethnicity were reported as unknown or not reported, treated as a separate category of variable, and were included in statistical testing. Missing data for family history and exposures were treated as if the patient did not have a family history or exposure for the purposes of analysis. No adjustment was made for multiple comparisons due to the exploratory nature of the investigation. Fisher’s exact test and Wilcoxon rank-sum test were used for comparison of categorical and continuous variables, respectively. Significance testing was not performed for data on other pregnancies as these were not independent observations.

## Results

**Figure 1:**
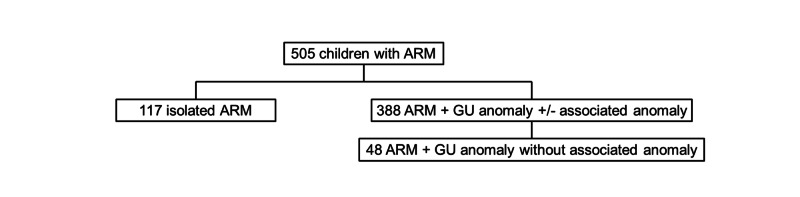
Flowchart of study population ARM: anorectal malformation; GU: genitourinary

A total of 505 patients (117 with isolated ARM and 388 with ARM and associated GU anomalies) were enrolled. Of the 388 patients with ARM and associated GU anomalies, 48 (12.4%) had an ARM with a GU anomaly and no anomaly of any other system (Figure 1). Only 10 of these 388 patients with associated GU anomalies had the GU anomalies identified prenatally.

In the GU-only cohort, the distribution of GU anomalies was as follows: 34 (70.8%) renal, 15 (31.3%) genital, 14 (29.2%) ureteral, four (8.3%) bladder, and two (4.2%) urethral. In the GU +/- additional anomalies cohort, the distribution of GU anomalies was as follows: 275 (70.9%) renal, 183 (47.2%) genital, 109 (28.1%) ureteral, 78 (20.1%) bladder, and 30 (7.7%) urethral (Table 1).

**Table 1:** Distribution of genitourinary malformations in the cohort of patients with ARM and an associated GU anomaly without additional anomalies (GU-only) and the cohort of patients with ARM and an associated GU anomaly with or without anomalies in other systems (GU +/- additional anomalies) ARM: anorectal malformation; GU: genitourinary

Malformation	GU-only	GU +/- additional anomalies	Malformation (continued)	GU-only	GU +/- additional anomalies
	(N = 48)	(N = 388)		(N = 48)	(N = 388)
Renal	34 (70.8%)	275 (70.9%)	Genital	15 (31.3%)	183 (47.2%)
Absent kidney	7 (14.6%)	53 (13.7%)	Absent testicle	0 (0%)	2 (0.5%)
Chronic kidney disease	2 (4.2%)	13 (3.4%)	Ambiguous genitalia	0 (0%)	6 (1.5%)
Cystic kidney	3 (6.3%)	19 (4.9%)	Absent uterus	0 (0%)	9 (2.3%)
Dysplastic kidney	2 (4.2%)	25 (6.5%)	Bicornate uterus	1 (2.1%)	13 (3.4%)
Ectopic/pelvic kidney	1 (2.1%)	16 (4.1%)	Cervical atresia/agenesis	0 (0%)	1 (0.3%)
Horseshoe kidney	2 (4.2%)	21 (5.4%)	Bifid scrotum	3 (6.3%)	19 (4.9%)
Hydronephrosis	23 (47.9%)	234 (60.3%)	Clitoromegaly	0 (0%)	2 (0.5%)
Kidney stones	2 (4.2%)	6 (1.5%)	Epispadias	0 (0%)	6 (1.5%)
Other	7 (14.6%)	63 (16.2%)	Hematocolpos	0 (0%)	2 (0.5%)
Bladder	4 (8.3%)	78 (20.1%)	Hydrocolpos	0 (0%)	10 (2.6%)
Bladder diverticulum	0 (0%)	9 (2.3%)	Penoscrotal transposition	1 (2.1%)	4 (1.0%)
Bladder exstrophy	0 (0%)	13 (3.4%)	Hypospadias	4 (8.3%)	38 (9.8%)
Bladder neck incompetence	0 (0%)	2 (0.5%)	Undescended testicle	3 (6.3%)	41 (10.6%)
Bladder neck obstruction	0 (0%)	1 (0.3%)	Unicornuate uterus	0 (0%)	1 (0.3%)
Bladder neck stricture	0 (0%)	1 (0.3%)	Uterine didelphys	3 (6.3%)	32 (8.2%)
Neurogenic bladder	2 (4.2%)	46 (11.9%)	Vaginal atresia	1 (2.1%)	17 (4.4%)
Other	2 (4.2%)	16 (4.1%)	Vaginal septum	4 (8.3%)	36 (9.3%)
Ureter	14 (29.2%)	109 (28.1%)	Other	4 (8.3%)	49 (12.6%)
Duplicated ureter	0 (0%)	10 (2.6%)	Urethra	2 (4.2%)	30 (7.7%)
Ectopic ureter	0 (0%)	6 (1.6%)	Diverticulum	0 (0%)	7 (1.8%)
Mega-ureter	0 (0%)	3 (0.8%)	Stenosis/ stricture	1 (2.1%)	15 (3.8%)
Ureterocele	0 (0%)	4 (1.0%)	Atresia	0 (0%)	2 (0.5%)
Ureteropelvic junction obstruction	1 (2.1%)	3 (0.8%)	Posterior urethral valves	1 (2.1%)	2 (0.5%)
Vesicoureteral reflux	14 (29.2%)	113 (29.1%)	Other	1 (2.1%)	7 (1.8%)
Other	2 (4.2%)	14 (3.6%)			

Of the patients in the GU +/- additional anomalies cohort, apart from GU anomalies, the most frequent associated anomalies were cardiovascular anomalies, which were present in 40.5% of patients in this cohort (Table 2).

**Table 2:** Associated anomalies of patients with ARM ARM: anorectal malformation; GU: genitourinary

Body system	GU +/- additional anomalies
	(N = 388)
Cardiovascular	
Minor	126 (32.5%)
Major	31 (8.0%)
Chromosomal	
Trisomy 21	14 (3.6%)
Other	24 (6.2%)
Endocrine	7 (1.8%)
Upper gastrointestinal	67 (17.3%)
Genital	183 (47.2%)
Craniofacial	40 (10.3%)
Hematologic	5 (1.3%)
Limb/extremity	28 (7.2%)
Neurologic	30 (7.7%)
Psychiatric	36 (9.3%)
Renal	275 (70.9%)
Bladder	78 (20.1%)
Ureter	109 (28.1%)
Urethra	30 (7.7%)
Respiratory	26 (6.7%)

Patient factors

There was a similar distribution of male and female patients in each cohort with no difference in the distribution of sex between the isolated ARM cohort and the GU-only or GU +/- additional anomalies cohorts. The majority of patients in all cohorts were whites and not Hispanic or Latino with no difference in the distribution of race or ethnicity between the cohorts. Patients in the GU +/- additional anomalies cohort were more likely to be born prematurely compared to the isolated ARM cohort (p=0.003). This difference was not statistically significant when comparing the GU-only cohort to the isolated ARM cohort. In addition, the patients in the GU +/- additional anomalies cohort had a lower birth weight compared to those in the isolated ARM cohort (2.9 vs 3.2 kg; p<0.001). This difference was not seen when comparing the GU-only cohort to the isolated ARM cohort (Table 3).

**Table 3:** Demographic data and birth outcomes of patients with ARM ARM: anorectal malformation; GU: genitourinary

	Isolated ARM	GU-only	p value	GU +/- additional anomalies	p value
	(N = 117)	(N = 48)		(N = 388)	
Gestational age at birth			0.16		0.003
Preterm (<37 weeks)	16 (13.7%)	10 (20.8%)		104 (26.8%)	
Term (>37 weeks)	74 (63.2%)	24 (50.0%)		216 (55.7%)	
Unknown	27 (23.1%)	14 (29.2%)		68 (17.5%)	
Birth weight (kg)	3.2 [2.8,3.5]	3.2 [2.6,3.7]	0.69	2.9 [2.4,3.3]	< 0.001
Sex			0.23		0.07
Male	53 (45.3%)	27 (56.3%)		214 (55.2%)	
Female	64 (54.7%)	21 (43.8%)		174 (44.8%)	
Race			0.20		0.85
White	74 (63.2%)	29 (60.4%)		249 (64.2%)	
Black/African American	16 (13.7%)	3 (6.3%)		46 (11.9%)	
Other	17 (14.5%)	13 (27.1%)		65 (16.8%)	
Unknown/not reported	10 (8.5%)	3 (6.3%)		28 (7.2%)	
Ethnicity			0.92		0.90
Hispanic or Latino	15 (12.8%)	7 (14.6%)		52 (13.4%)	
Not Hispanic or Latino	99 (84.6%)	40 (83.3%)		328 (84.5%)	
Unknown/not reported	3 (2.6%)	1 (2.1%)		8 (2.1%)	

Family history

There was a low prevalence of family history of ARM in all three cohorts with no statistical difference in this prevalence between the cohorts. In addition, there was no difference in the prevalence of family history of colorectal cancer, Hirschsprung's disease, inflammatory bowel disease, motility disorders, other congenital anomalies, or chromosomal anomalies between the cohorts. The total number of pregnancies other than the patient was 66, 60, and 482 in the isolated ARM, GU-only, and GU +/- additional anomalies cohorts, respectively. The majority of these were singleton pregnancies resulting in live births in all cohorts (Table 4).

**Table 4:** Family history of patients with ARM ARM: anorectal malformation; GU: genitourinary

	Isolated ARM	GU-only	p value	GU +/- additional anomalies	p value
	(N = 117)	(N = 48)		(N = 388)	
ARM			0.87		0.74
Paternal					
Father	0 (0%)	0 (0%)		0 (0%)	
Relative	2 (1.7%)	0 (0%)		1 (0.3%)	
Maternal					
Mother	0 (0%)	0 (0%)		0 (0%)	
Relative	0 (0%)	0 (0%)		0 (0%)	
Sibling	0 (0%)	1 (2.1%)		4 (1.0%)	
Colorectal cancer	2 (1.7%)	2 (4.2%)	0.58	13 (3.4%)	0.54
Hirschsprung's disease	0 (0%)	0 (0%)	-	0 (0%)	-
Inflammatory bowel disease	1 (0.9%)	2 (4.2%)	0.20	12 (3.1%)	0.32
Motility disorder	1 (0.9%)	1 (2.1%)	0.50	3 (0.8%)	1.00
Other congenital anomalies	3 (2.6%)	2 (4.2%)	0.63	7 (1.8%)	0.70
Chromosomal abnormality	1 (0.9%)	0 (0%)	1.00	1 (0.3%)	0.41
Other maternal pregnancies	66	60		482	
Multiple pregnancies, e.g., twins					
No	56 (84.8%)	51 (85.0%)		366 (75.9%)	
Yes	4 (6.1%)	3 (5.0%)		35 (7.3%)	
Unknown	6 (9.1%)	6 (10.0%)		81 (16.8%)	
Pregnancy outcome					
Live birth	61 (92.4%)	60 (100%)		464 (96.3%)	
Miscarriage	4 (6.1%)	0 (0%)		10 (2.1%)	
Termination of pregnancy	1 (1.5%)	0 (0%)		8 (1.7%)	

Maternal exposures and comorbidities

There was no difference in the prevalence of alcohol, tobacco, or drug exposure during pregnancy between the cohorts. The most frequent maternal comorbidity in all cohorts was gestational diabetes, which was present in 6-7% of mothers in all cohorts. There was no difference between the cohorts in the prevalence of any maternal comorbidity (Table 5).

**Table 5:** Frequency of maternal exposures/comorbidities and prenatal conditions in patients with ARM ^*^Other comorbidities occurred in two or less mothers ARM: anorectal malformation; GU: genitourinary

	Isolated ARM	GU-only	p value	GU +/- additional anomalies	p value
	(N = 117)	(N = 48)		(N = 388)	
Substance exposures					
Alcohol exposure	5 (4.3%)	2 (4.2%)	1.00	5 (1.3%)	0.57
Tobacco exposure	5 (4.3%)	0 (0%)	0.32	10 (2.6%)	0.36
Recreational drug exposure	6 (5.1%)	1 (2.1%)	0.68	9 (2.3%)	0.13
Comorbidities					
Gestational diabetes	9 (7.7%)	3 (6.3%)	1.00	25 (6.4%)	0.67
Hypertension	5 (4.3%)	0 (0%)	0.15	6 (1.5%)	0.08
Preeclampsia	1 (0.9%)	0 (0%)	0.52	5 (1.3%)	0.06
In-vitro fertilization	1 (0.9%)	0 (0%)	1.00	5 (1.3%)	1.00
Insulin-dependent diabetes	2 (1.7%)	0 (0%)	1.00	1 (0.3%)	0.14
Other^*^	5 (4.3%)	1 (2.1%)	0.50	21 (5.4%)	0.63
Prenatal conditions					
Intrauterine growth retardation	4 (3.4%)	0 (0%)	0.32	12 (3.1%)	0.77
Polyhydramnios	1 (0.9%)	1 (2.1%)	0.50	18 (4.6%)	0.09
Oligohydramnios	2 (1.7%)	0 (0%)	1.00	13 (3.4%)	0.54
Multiparous gestation	1 (0.9%)	1 (2.1%)	0.50	9 (2.3%)	0.47

Prenatal conditions

The most prevalent prenatal conditions in the isolated ARM cohort were intrauterine growth retardation (3.4%) and oligohydramnios (1.7%). The most prevalent prenatal conditions in the GU-only cohort were polyhydramnios (2.1%) and multiparous gestation (2.1%). The most prevalent prenatal conditions in the GU +/- additional anomalies cohort were polyhydramnios (4.6%) and oligohydramnios (3.4%). There were no differences in the prevalence of these prenatal conditions between the cohorts (Table 5).

## Discussion

This is the first report comparing patients with isolated ARM to those with ARM and associated GU anomalies from the PCPLC. We identified that patients with ARM and an associated GU malformation with or without an anomaly in another system were more likely to be born prematurely compared to patients with isolated ARM. However, this significant difference in preterm birth was not present in patients with ARM and an associated GU malformation only. This may be due to the lower sample size of this cohort. However, this difference between the GU +/- additional anomalies and GU-only cohorts regarding preterm birth could also be due to other congenital anomalies, such as cardiac anomalies, driving the tendency toward prematurity in the GU +/- additional anomalies cohort. However, cardiac anomalies seem unlikely to be the sole cause of this difference because, if you compare the prevalence of prematurity in patients with cardiac anomalies to those without cardiac anomalies in our population, there is no statistical difference (35.7 vs 28.3%; p=0.16). Furthermore, this difference could be driven by the differences in the types of GU malformations present in each cohort. Specifically, there was a much higher percentage of bladder anomalies in the GU +/- additional anomalies cohort (20.1%) compared to the GU-only cohort (8.3%). Looking at the breakdown of these bladder anomalies, half of these anomalies in the GU +/- additional anomalies cohort were neurogenic bladder. It is unlikely that neurogenic bladder alone would play a significant role in prematurity, and this is likely related to the collinear nature of neurogenic bladder and the presence of other associated anomalies. For example, neurogenic bladder in ARM is often related to a spinal cord abnormality. These patients would, by definition, have other associated anomalies in other systems. More research on this topic is necessary to help elucidate these finer associations and to determine what is actually driving this difference between these cohorts. In the literature, there are mixed data on the association between any patient with ARM and preterm birth [3,5,6]. Vermes et al. compared the birth outcomes of patients with isolated ARM to children without congenital anomalies and found that there was no difference in the prevalence of preterm births [15]. Variable reports regarding the prevalence of preterm birth in ARM may be a result of combining those with an isolated ARM and all patients with an ARM, as we identified a significantly higher prevalence of preterm births in patients with ARM and associated GU abnormalities compared to isolated ARM. In light of our findings, it seems reasonable to counsel parents of a fetus with an ARM and associated GU anomalies in the setting of other congenital anomalies on the risk of preterm delivery and prepare accordingly.

Additionally, this study illustrates the benefit of utilizing a consortium to study these rare pediatric colorectal and pelvic diseases. Over a 29-month study period, the PCPLC enrolled 505 patients (189 enrolled per year) with ARM. This rate of enrollment is significantly higher than most non-consortium-based studies of patients with ARM [6,9,12,15,16]. Previous studies of ARM by Svenningsson et al., Zwink et al., and de Blaauw et al. have also utilized consortia or national registries to increase the rate of enrollment [5,7,17]. Zwink et al. utilized the German Network for Congenital Uro-REctal malformations (CURE-Net) and de Blaauw et al. utilized the European Consortium on Anorectal Malformations (ARM-Net), which is a conglomeration of European national registries of ARM patients, including the German CURE-Net network [7,17]. Like the PCPLC, this consortium enrolls significantly more patients than non-consortium-based studies, with de Blaauw et al. enrolling almost 41 patients per year [17]. However, the PCPLC is unique, in that the database represents multiple centers in a single country rather than limited centers of expertise across multiple countries as is the structure of ARM-Net. In contrast, the Swedish National Patient Register utilized by Svenningsson et al. similarly allowed for the analysis of a significant number of patients with ARM due to the prolonged period of time over which data were collected. Like the PCPLC, this register captures multiple institutions in a single country. However, despite the large sample size, Svenningsson et al. enrolled 28 patients per year, which is closer to previous single-institution studies and limits the growth of the cohort and generalizability of the data, given the historical nature of the cohort [5]. Neither of these other consortia has utilized their database to compare isolated ARM to ARM with other anomalies, such as GU anomalies, so there is no direct comparison that can be drawn between our results and those from ARM-Net or the Swedish National Patient Register. Interestingly, Svenningsson et al. compared patients with ARM to healthy controls and found that patients with ARM were 2.7 times more likely to be born preterm and 4.8 times more likely to be born very preterm. However, the authors also compared the subset of patients with isolated ARM to healthy controls and found that patients with ARM were two times more likely to be born preterm and 3.3 times more likely to be born very preterm [5]. Given that the effect size decreased with the removal of the patients with associated congenital abnormalities, it is possible that the Swedish National Patient Register would show similar findings to ours where an equivalent analysis was performed.

This study has multiple limitations. The retrospective nature of this study increases the risk of confounding factors and bias, specifically recall bias, from mothers regarding substance exposures. Furthermore, the timing of prenatal substance exposures during pregnancy is unknown. It is unlikely that an exposure in the third trimester would contribute to ARM, thus the exposure may be overstated. In addition, this study compared isolated ARM to ARM with associated GU anomalies but did not have a non-ARM comparator group. Therefore, we cannot identify the risk factors of ARM, and can only identify its associations.

## Conclusions

Patients with ARM and an associated GU anomaly with or without congenital anomalies in other systems are more likely to be born prematurely compared to patients with an isolated ARM. Parents of these children should be counseled on this increased risk. Furthermore, utilization of a multi-institutional consortium in studying rare pediatric colorectal diseases, such as ARM, allows for the rapid enrollment of a significant number of patients in a short period of time.

## References

[1] Falcone RA, Levitt MA, Peña A, Bates M (2007). Increased heritability of certain types of anorectal malformations. J Pediatr Surg.

[2] Marcelis C, de Blaauw I, Brunner H (2011). Chromosomal anomalies in the etiology of anorectal malformations: A review. Am J Med Genet A.

[3] Wijers CH, van Rooij IA, Marcelis CL, Brunner HG, de Blaauw I, Roeleveld N (2014). Genetic and nongenetic etiology of nonsyndromic anorectal malformations: A systematic review. Birth Defects Res C Embryo Today Rev.

[4] Wang C, Li L, Cheng W (2015). Anorectal malformation: the etiological factors. Pediatr Surg Int.

[5] Svenningsson A, Gunnarsdottir A, Wester T (2018). Maternal risk factors and perinatal characteristics of anorectal malformations. J Pediatr Surg.

[6] van Rooij IA, Wijers CH, Rieu PN (2010). Maternal and paternal risk factors for anorectal malformations: a Dutch case-control study. Birth Defects Res A Clin Mol Teratol.

[7] Zwink N, Rissmann A, Pötzsch S, Reutter H, Jenetzky E, CURE-Net Consortium (2016). Parental risk factors of anorectal malformations: Analysis with a regional population-based control group. Birth Defects Res A Clin Mol Teratol.

[8] Wijers CH, de Blaauw I, Marcelis CL (2010). Research perspectives in the etiology of congenital anorectal malformations using data of the International Consortium on Anorectal Malformations: evidence for risk factors across different populations. Pediatr Surg Int.

[9] Wijers CH, van Rooij IA, Rassouli R (2015). Parental subfertility, fertility treatment, and the risk of congenital anorectal malformations. Epidemiology.

[10] van de Putte R, Wijers CH, de Blaauw I (2017). Previous miscarriages and GLI2 are associated with anorectal malformations in offspring. Hum Reprod.

[11] Zwink N, Jenetzky E (2018). Maternal drug use and the risk of anorectal malformations: systematic review and meta-analysis. Orphanet J Rare Dis.

[12] van den Hondel D, Wijers CH, van Bever Y (2016). Patients with anorectal malformation and upper limb anomalies: genetic evaluation is warranted. Eur J Pediatr.

[13] Khanna K, Sharma S, Pabalan N, Singh N, Gupta DK (2018). A review of genetic factors contributing to the etiopathogenesis of anorectal malformations. Pediatr Surg Int.

[14] Reeder RW, Wood RJ, Avansino JR (2018). The Pediatric Colorectal and Pelvic Learning Consortium (PCPLC): rationale, infrastructure, and initial steps. Tech Coloproctol.

[15] Vermes G, László D, Mátrai Á, Czeizel AE, Ács N (2015). Maternal factors in the origin of isolated anorectal malformations - a population-based case-control study. J Matern Fetal Neonatal Med.

[16] Yuan P, Okazaki I, Kuroki Y (1995). Anal atresia: Effect of smoking and drinking habits during pregnancy. Jpn J Hum Genet.

[17] de Blaauw I, Wijers CH, Schmiedeke E (2013). First results of a European multi-center registry of patients with anorectal malformations. J Pediatr Surg.

